# Dietary Aflatoxin B1 attenuates immune function of immune organs in grass carp (Ctenopharyngodon idella) by modulating NF-κB and the TOR signaling pathway

**DOI:** 10.3389/fimmu.2022.1027064

**Published:** 2022-10-18

**Authors:** Xiang-Ning He, Zhen-Zhen Zeng, Pei Wu, Wei-Dan Jiang, Yang Liu, Jun Jiang, Sheng-Yao Kuang, Ling Tang, Lin Feng, Xiao-Qiu Zhou

**Affiliations:** ^1^ Animal Nutrition Institute, Sichuan Agricultural University, Chengdu, China; ^2^ Fish Nutrition and Safety Production University Key Laboratory of Sichuan Province, Sichuan Agricultural University, Chengdu, China; ^3^ Key Laboratory for Animal Disease-Resistance Nutrition, Ministry of Education, Ministry of Agriculture and Rural Affairs, Key Laboratory of Sichuan Provence, Chengdu, China; ^4^ Animal Nutrition Institute, Sichuan Academy of Animal Science, Chengdu, China

**Keywords:** AFB1, spleen, head kidney, skin, immune

## Abstract

Aflatoxin B1 (AFB1) is kind of a common mycotoxin in food and feedstuff. Aquafeeds are susceptible to contamination of AFB1. In teleost fish, the spleen and head kidney are key immune organ. Moreover, the fish skin is a critical mucosal barrier system. However, there was little study on the effects of dietary AFB1 on the immune response of these immune organs in fish. This study aimed to explore the impacts of oral AFB1 on the immune competence and its mechanisms in the skin, spleen, and head kidney of grass carp. Our work indicated that dietary AFB1 reduced antibacterial compounds and immunoglobulins contents, and decreased the transcription levels of antimicrobial peptides in grass carp immune organs. In addition, dietary AFB1 increased the transcription levels of pro-inflammatory cytokines and reduced the transcription levels of anti-inflammatory cytokines in the grass carp immune organs, which might be regulated by NF-κB and TOR signaling, respectively. Meanwhile, we evaluated the content of AFB1 in the grass carp diet should not exceed 29.48 μg/kg diet according to the levels of acid phosphatase and lysozyme. In summary, dietary AFB1 impaired immune response in grass carp skin, spleen, and head kidney.

## Introduction

As the plant raw materials gradually in place of animal raw materials, the contamination of agriculture products by mycotoxin is a worldwide concern problem, which caused a problem of feed safety, including aquafeed (1). Aflatoxin B1 (AFB1) is a kind of common mycotoxin, which could induce inflammatory response in mutton sheep muscle (2). It was found that AFB1 could be detected in fish feed in most areas, and up to 150 μg/kg (3). Fish that consume AFB1-contaminated feed experience detrimental health effects, and even endanger human health (4). The only study found that AFB1 induced intestinal inflammation by upregulating *TNF-α* and *IL-1β* mRNA expression in rainbow trout (5). Previous research demonstrated that AFB1 stunted the growth of grass carp (6). It has been known that the growth of fish is associated with its immunity (7). As central immune organs in teleost fish, the spleen and head kidney are responsible for regulating immune response (8), containing large numbers of lymphocytes and macrophages (9, 10). As we all know, lymphocytes and macrophages are an essential part of the immune system (11). The fish’s skin, an important mucosal defense organ, has developed a mature immune barrier to protect the whole body from pathogens invasion (12, 13). Currently, there is no study has been conducted on how AFB1 affects fish skin, spleen, and head kidney immunity. According to a previous report, organs’ function is closely related to their structural integrity (14). Huang et al. (15) study found that deoxynivalenol (DON) damaged the structural integrity of grass carp intestinal, which resulted in a decrease in the intestinal immune function (16). Another previous research exhibited that AFB1 disrupted the structure of the spleen and head kidney, causing tissue vacuolar degeneration, melanoma macrophage center, and cell necrosis (6). The above studies indicate that the reduction of fish immunity by AFB1 may be related to the destruction of mucosal immunity and central immune, which is worthy of further study.

The spleen and head kidney are the heart of systemic immunity, which are involved in the process of systemic immune response (17, 18). When inflammation occurs, the spleen and head kidney usually respond by secreting antimicrobial substances and cytokines (19). It is well known that skin serves as the first line of defense against invading pathogens (12). It has been shown that in fish skin, goblet cells and lymphocytes secrete antibacterial compounds and immunoglobulin (20). In addition, skin is a crucial immunological organ in fish, which forms a better immune barriers system to protect the body from attacks. Our previous studies found that obvious yellowing of surface was observed on grass carp skin with the increasing AFB1 concentration. We hypothesized that AFB1 might aggravate inflammation in grass carp skin. However, there is no report about the effect of AFB1 on antibacterial compounds and cytokines in the skin, spleen, and head kidney of fish. Yang et al. (21) found that AFB1 decreased the immune parameters (e.g. IgM, C3, and C4) in juvenile turbot serum. In addition, AFB1 decreased the viability and induced an inflammatory response in macrophages (22). Meanwhile, one study found that AFB1 upregulated the gene levels of *IL-1β* in the intestine mucosa of the broiler (23), but another study suggested that dietary AFB1 did not change *IL-1β* gene expression in sheep muscle (2). The above results indicated that different animal tissues have different immune responses to AFB1. However, beyond this, Guo et al. (24) found that AFB1 upregulated significantly the mRNA levels of pro-inflammatory cytokines *IL-6*, *IL-8*, and *TNF-α* in kidney cells of chicken embryos. And Cao et al. (2) found that AFB1 downregulated the mRNA level of anti-inflammatory cytokine *IL-10* in mutton sheep muscle. Studies conducted above indicated that AFB1 could induce inflammation in animals. However, there is no relevant report on the immune response of AFB1 to fish skin, spleen, and head kidney. So it is necessary to study the impacts of AFB1 on the immune competence of the skin, spleen, and head kidney of fish.

It is well known that an important transcription factor nuclear factor kappa B p65 (NF-κB p65) is involved in inflammatory responses (25). When the NF-κB is activated, it increases the transcription of proinflammatory cytokines (26). It has not been reported that AFB1 regulates the immune response of fish immune organs through NF-κB and target of rapamycin (TOR) signaling pathways. It was reported that AFB1 activated NF-κB signaling pathways and induced inflammatory responses in ducks’ ileum (27). Another study found that AFB1 upregulated *NF-κB p65* mRNA expression and induced inflammatory responses in chicken embryos’ primary intestinal epithelium (24). These results indicated that AFB1 could induce inflammatory responses in animals by activating the NF-κB signaling pathway. Furthermore, the TOR pathway has a significant role to play in immunoregulation (28). It was reported that anti-inflammatory cytokines in the skin, spleen, and head kidney of fish are regulated by the TOR signaling pathway (12, 29). Ivanovics et al. (30) found that AFB1 exposure significantly reduced the arginine level in zebrafish larvae lysates. Chen et al. (31) reported that arginine deficiency downregulated TOR mRNA expression in Jian carp (*Cyprinus carpio* var. *Jian*) spleen and head kidney. Following the above reports, we speculate that AFB1 might induce inflammatory responses through NF-κB p65 and mTOR signaling pathways, which needs further in‐depth studies.

Grass carp is a kind of important economic freshwater fish in China, accounting for more than 18% of total freshwater aquaculture production in 2017 (32). It is well known that grass carp belong to herbivorous fish, it is usually with plant protein raw materials in the majority of commercial feed (33). Thus, in the process of large-scale commercial production of fish feed, it is very likely that there is a problem with excess AFB1 residue. Our research has focused on the immune components and cytokines and its relative signaling of grass carp skin, spleen, and head kidney. Furthermore, we evaluated the AFB1 safe upper dose for grass carp according to the ACP and LZ, to provide a practical reference for production.

## Materials and methods

### Composition of test feeds

The feed formulation was identical to our previous research (6). A basal experimental diet was prepared using casein, gelatin, and fish meal as protein sources, soybean oil, and fish oil as lipid sources. All feed ingredients contain no AFB1. The relative diet’s nutrition levels met the growing need for juvenile grass crap. AFB1 (purity > 98%) was bought from Pribolab Pte. Ltd. (Singapore). There were six different concentrations [0 (un-supplement), 30, 60, 90, 120, and 150 μg/kg diet, respectively] of AFB1 in the feed. We used high-performance liquid chromatographic (HPLC) to measure the actual concentration, which were 0.04 (un-supplement), 29.48, 58.66, 85.94, 110.43 and 146.92 μg/kg diet, respectively.

### Feeding trial

The use of animals in experiments was under the regulations established by the Sichuan Agricultural University Animal Care Advisory Committee. After purchase, the grass carp were adapted to the environment for 28 days before the experiment. The average weight of the grass carp was 12.96 ± 0.03 g. They were randomly allocated into 6 groups of three replicated cages. Our experiment was conducted outdoor. The average water temperature is 28.5 ± 2°C. The pH value was around 7.5 ± 0.3. We maintained the dissolved oxygen in water > 6.0 mg/L by adjusting the oxygenator. The experiment was carried out under a natural light cycle.

### Challenge test and sample collection

The grass carp was challenged with *A. hydrophila* for 2 weeks after the growth experiment. *A. hydrophila* infection is a serious problem in aquaculture (34), which is usually used to evaluate the effect on the immunity of fish (35). Our *A. hydrophila* was generously gifted by the College of Veterinary Medicine, Sichuan Agricultural University. 15 fish were treated with an intraperitoneal injection of *A. hydrophila* (1.0 *ml*, 2.5x10^6^ colony-forming units (CFU)/ml) for every treatment. And the control group was infected with 1.0 ml saline.

All the fish were anesthetized with benzocaine (50 mg/L) after the challenge test. The samples were selected and flash frozen on liquid nitrogen, a portion of the samples was quickly frozen in a -20°C freezer for the analysis of immune components, and the other part of the samples were removed in a -80°C freezer for PCR and Western blot analysis.

### Immune components analysis

The sample preparation for immune components determination was referenced by Lu et al. (12). The immunoglobulin M (IgM), ACP, and Lysozyme (LZ) activity, complement component 3 (C3), and component 4 (C4) contents were tested by assay kit (Nanjing Jiancheng, Chain). According to the kit instructions, the specific operation was performed.

### Real-time PCR

The RNAiso Plus kit (TaKaRa, China) was used to extract total RNA. RNA concentrations were obtained by spectrophotometry, and RNA quality was confirmed by agarose gel electrophoresis (1%). Then, cDNA synthesis was performed with a cDNA synthesis Kit (Takara, China). The quantitative real-time PCR primers were indicated in **Table 1**. The β-actin was used as an internal reference gene.

**Table 1:** Characteristics of different expression genes related to immune function.

Target gene	Primer sequence Forward (5’→3’)	Primer sequence Reverse (5’→3’)	Temperature(°C)	Accession number
***hepcidin***	AGCAGGAGCAGGATGAGC	GCCAGGGGATTTGTTTGT	59.3	JQ246442.1
***LEAP-2A***	TGCCTACTGCCAGAACCA	AATCGGTTGGCTGTAGGA	59.3	FJ390414
***LEAP-2B***	TGTGCCATTAGCGACTTCTGAG	ATGATTCGCCACAAAGGGG	59.3	KT625603
***β-defensin-1***	TTGCTTGTCCTTGCCGTCT	AATCCTTTGCCACAGCCTAA	58.4	KT445868
***Mucin2***	GAGTTCCCAACCCAACACAT	AAAGGTCTACACAATCTGCCC	60.4	KT625602
***IFN-γ2***	TGTTTGATGACTTTGGGATG	TCAGGACCCGCAGGAAGAC	60.4	JX657682
***TNF-α***	CGCTGCTGTCTGCTTCAC	CCTGGTCCTGGTTCACTC	58.4	HQ696609
***IL-1β***	AGAGTTTGGTGAAGAAGAGG	TTATTGTGGTTACGCTGGA	57.1	JQ692172
***IL-6***	CAGCAGAATGGGGGAGTTATC	CTCGCAGAGTCTTGACATCCTT	62.3	KC535507.1
***IL-8***	ATGAGTCTTAGAGGTCTGGGT	ACAGTGAGGGCTAGGAGGG	60.3	JN663841
***IL-10***	AATCCCTTTGATTTTGCC	GTGCCTTATCCTACAGTATGTG	61.4	HQ388294
***IL-12p35***	TGGAAAAGGAGGGGAAGATG	AGACGGACGCTGTGTGAGTGTA	55.4	KF944667.1
***IL-12p40***	ACAAAGATGAAAAACTGGAGGC	GTGTGTGGTTTAGGTAGGAGCC	59.0	KF944668.1
***IL-15***	CCTTCCAACAATCTCGCTTC	AACACATCTTCCAGTTCTCCTT	61.4	KT445872
***IL-17D***	GTGTCCAGGAGAGCACCAAG	GCGAGAGGCTGAGGAAGTTT	62.3	KF245426.1
***IL-4/13A***	CTACTGCTCGCTTTCGCTGT	CCCAGTTTTCAGTTCTCTCAGG	55.9	KT445871
***IL-4/13B***	TGTGAACCAGACCCTACATAACC	TTCAGGACCTTTGCTGCTTG	55.9	KT625600
***TGF-β1***	TTGGGACTTGTGCTCTAT	AGTTCTGCTGGGATGTTT	55.9	EU099588
***TGF-β2***	TACATTGACAGCAAGGTGGTG	TCTTGTTGGGGATGATGTAGTT	55.9	KM279716
***NF-κB p52***	TCAGTGTAACGACAACGGGAT	ATACTTCAGCCACACCTCTCTTAG	58.4	KM279720
***NF-κB p65***	GAAGAAGGATGTGGGAGATG	TGTTGTCGTAGATGGGCTGAG	62.3	KJ526214
***c-Rel***	GCGTCTATGCTTCCAGATTTACC	ACTGCCACTGTTCTTGTTCACC	59.3	KT445865
***IκBα***	TCTTGCCATTATTCACGAGG	TGTTACCACAGTCATCCACCA	62.3	KJ125069
***IKKα***	GGCTACGCCAAAGACCTG	CGGACCTCGCCATTCATA	60.3	KM279718
***IKKβ***	GTGGCGGTGGATTATTGG	GCACGGGTTGCCAGTTTG	60.3	KP125491
***IKKγ***	AGAGGCTCGTCATAGTGG	CTGTGATTGGCTTGCTTT	58.4	KM079079
***TOR***	TCCCACTTTCCACCAACT	ACACCTCCACCTTCTCCA	61.4	JX854449
***S6K1***	TGGAGGAGGTAATGGACG	ACATAAAGCAGCCTGACG	54.0	EF373673
***4E-BP1***	GCTGGCTGAGTTTGTGGTTG	CGAGTCGTGCTAAAAAGGGTC	60.3	KT757305
***4E-BP2***	TGTGCCATTAGCGACTTCTGAG	ATGATTCGCCACAAAGGGG	59.3	KT625603
***β-actin***	GGCTGTGCTGTCCCTGTA	GGGCATAACCCTCGTAGAT	61.4	M25013

### Western blot

An aliquot of the homogenates was used for protein measurement, the method was performed as our lab method (36). For each sample, total proteins (40 μg) were extracted and separated on sodium dodecyl sulfate-polyacrylamide gel electrophoresis (8%) and then transferred to polyvinylidene-fluoride membrane immunoblotting membranes. The membranes were incubated overnight in the primary antibody at 4°C. Then the membranes were washed and incubated with the secondary antibodies at room temperature for 90 min. Immunocomplexes were visualized by enhanced chemiluminescence reagents (Beyotime Biotechnology Inc., China), then quantifications were performed in Image 1.63 software. The information of antibodies was listed in **Supplementary Table 1**.

### Statistical analysis

All data were represented as the mean ± standard deviation (SD). Significance differences among groups were determined by using one-way analysis of variance (ANOVA). The significant difference among the different groups’ means was tested by using Duncan’s multiple ranges, and data were analyzed by SPSS 18.0 (SPSS Inc., Chicago, IL, USA). The experiment data were visualized by GraphPad 8.0 software and the Hiplot platform (12).

## Results

### Skin phenotype

**Figure 1:**
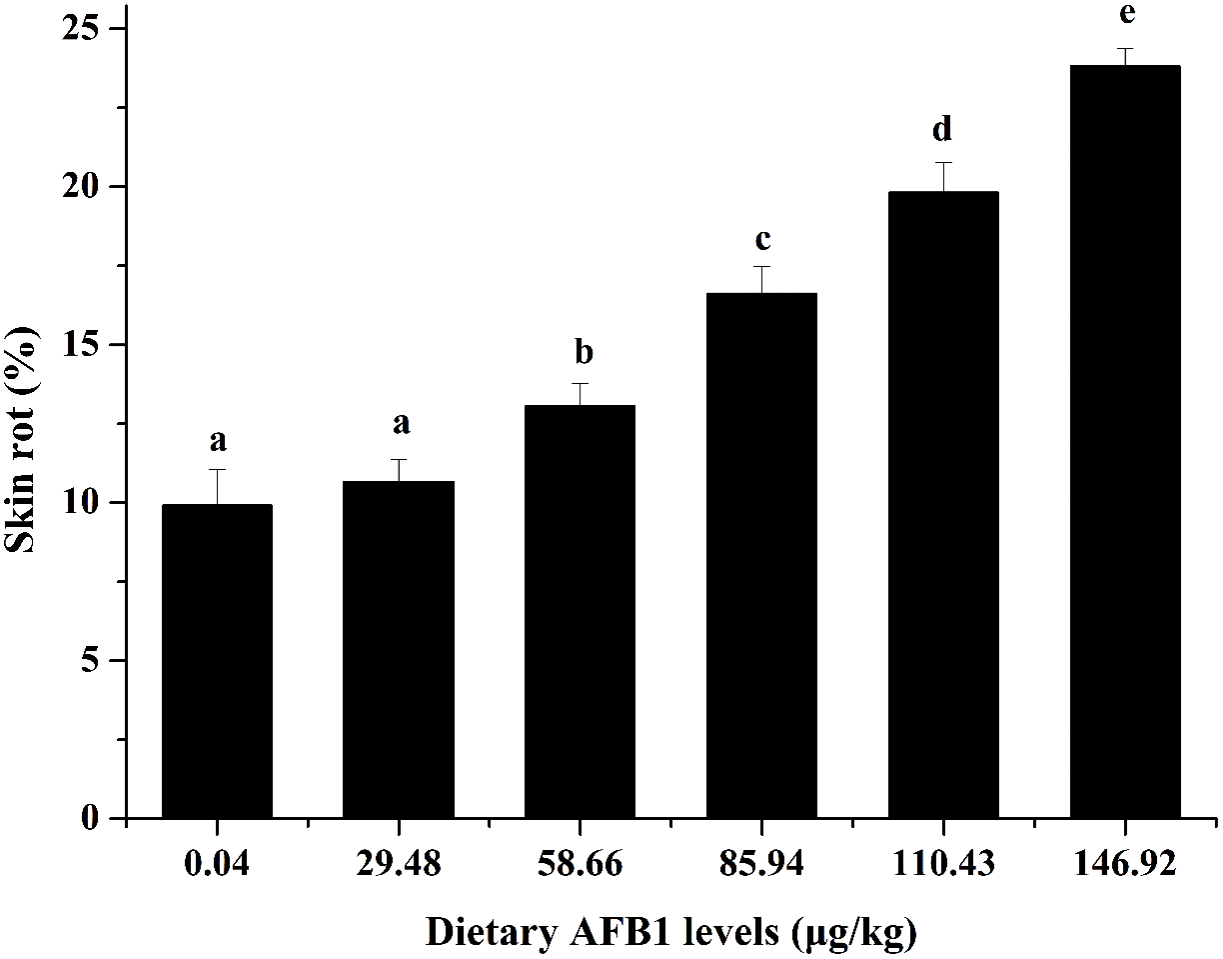
Effects of dietary AFB1 (μg/kg) on skin rot of grass carp after challenged with *A. hydrophila*. n=6 (six fish in each group). Different letters indicate significant differences (P < 0.05).

It is well known that skin rot is the typical symptom of *Aeromonas hydrophila* infection in fish. Our previous study found that obvious yellowing of surface were observed on grass carp skin with the increasing AFB1 concentration, which is the typical symptom of skin rot (6). As shown in **Figure 1**, compared to the control group (0.04 μg/kg diet), the skin rot was increased with the increase of AFB1 (58.66-146.92 μg/kg diet), and showed a highly positive correlation.

### Immune parameters in skin, spleen, and head kidney of grass carp

**Figure 2:**
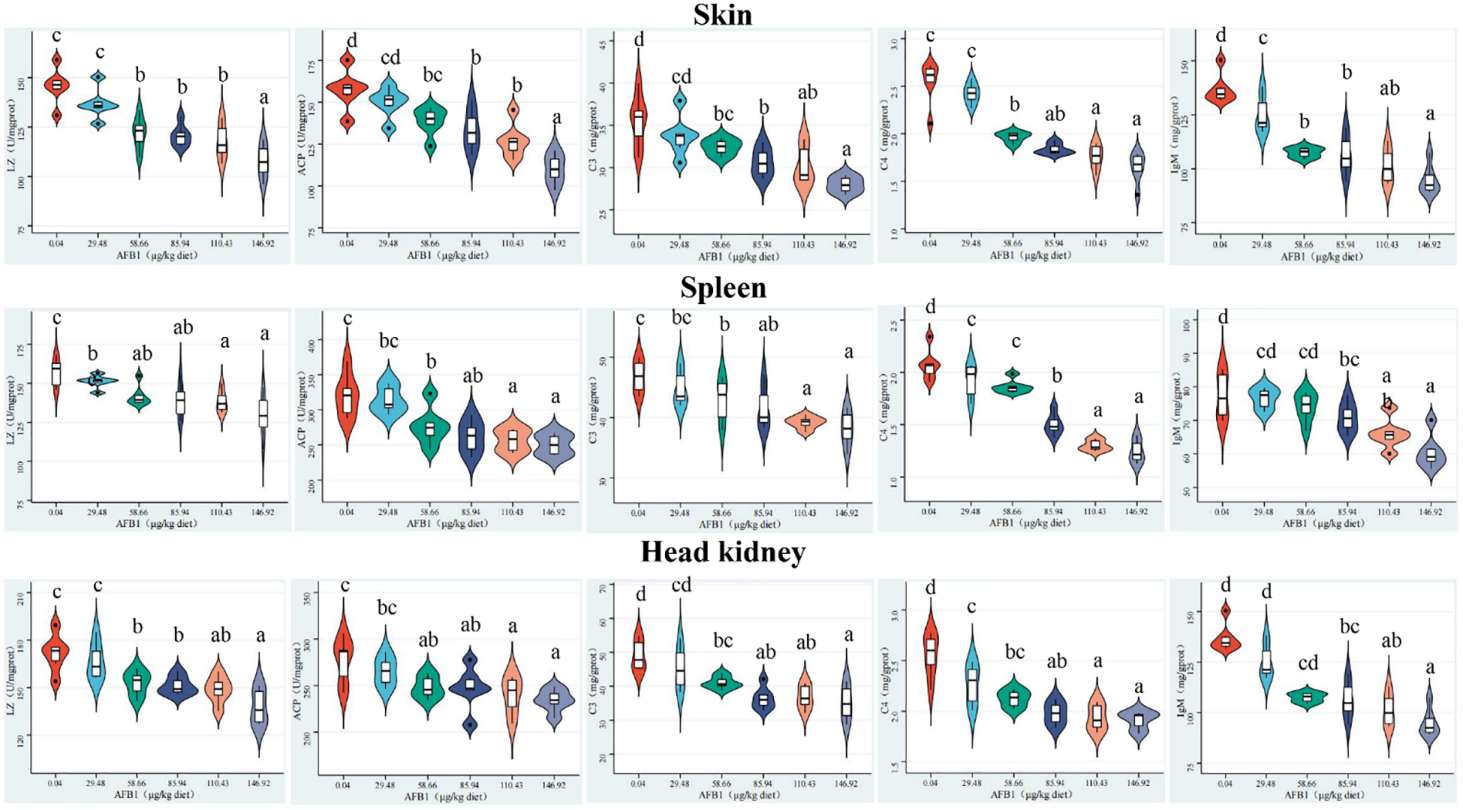
Effects of dietary AFB1 (μg/kg) on the immune parameters (LZ, ACP, C3, C4, IgM) of skin, spleen, and head kidney of grass carp after challenged with *A. hydrophila*. n=6 (six fish in each group). Different letters represent a significance difference (P < 0.05).

The immune-related parameters of skin, spleen, and head kidney were given in **Figure 2**. In the skin, the C3, C4, and IgM content and the LZ, ACP activities were reduced when the dose of AFB1 reached 58.66, 58.66, 29.48, 58.66, and 58.66 μg/kg diet (*P* < 0.05), respectively. But in the spleen and head kidney, the above immune parameters were decreased with the level of AFB1 up to 58.66, 29.48, 85.94, 58.66, and 58.66 μg/kg diet (*P* < 0.05), respectively. The immune components reached the lowest level in the 146.92 μg/kg diet groups in the immune organs of grass carp.

### The transcription levels of antimicrobial peptides and mucin in skin, spleen, and head kidney of grass carp

**Figure 3:**
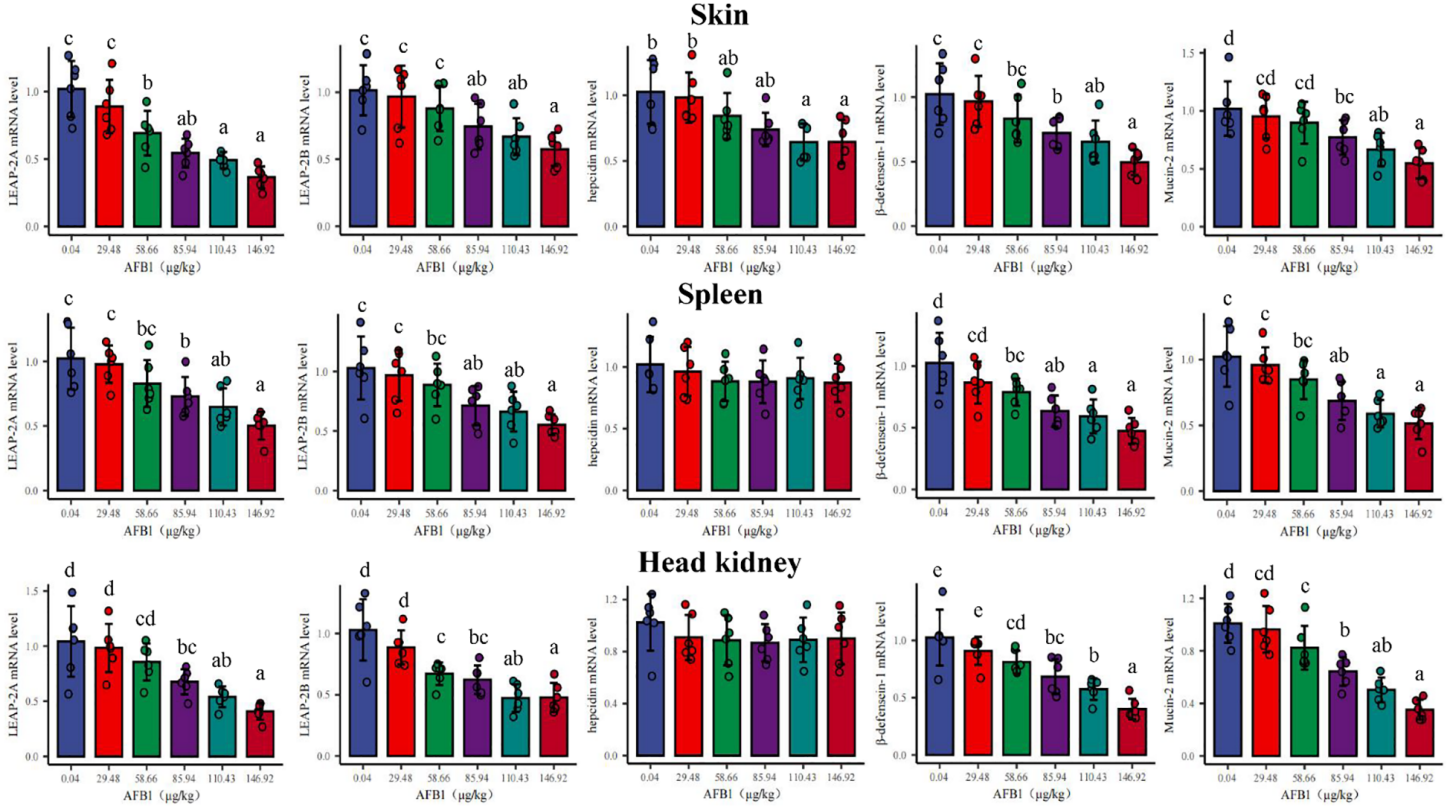
Effects of dietary AFB1 (μg/kg) on the mRNA levels of antibacterial peptides (*LAEP-2A*, *LEAP-2B*, *hepcidin*, *β-defenscin-1*) and *Mucin-2* of skin, spleen, and head kidney of grass carp after challenged with *A. hydrophila*. n=6 (six fish in each group). Different letters represent a significance difference (P < 0.05).

In **Figure 3**, in the skin, the gene expression of *hepcidin*, *β-defensin-1*, *LEAP-2A*, *Mucin2*, and *LEAP-2B* were significantly downregulated with the dose of AFB1 up to 85.94, 85.94, 58.66, 85.94, and 85.94μg/kg diet (*P* < 0.05), respectively. In the spleen, there was a significantly decreased in *β-defensin-1*, *LEAP-2A*, *Mucin2*, and *LEAP-2B* gene expression when the dietary AFB1 dose was up to 58.66, 85.94, 85.94, and 85.94 μg/kg diet (*P* < 0.05), respectively. In the head kidney, the gene expression of *β-defensin-1*, *LEAP-2A*, *Mucin2*, and *LEAP-2B* were significantly decreased when the dose of AFB1 reached 58.66, 85.94, 58.66, and 58.66 μg/kg diet (*P* < 0.05), respectively. Interestingly, in the spleen and head kidney, *hepcidin* mRNA expression had not changed at any level of AFB1.

### The transcription levels of inflammatory cytokines and inflammatory response-related signal molecules in skin, spleen, and head kidney of grass carp

**Figure 4:**
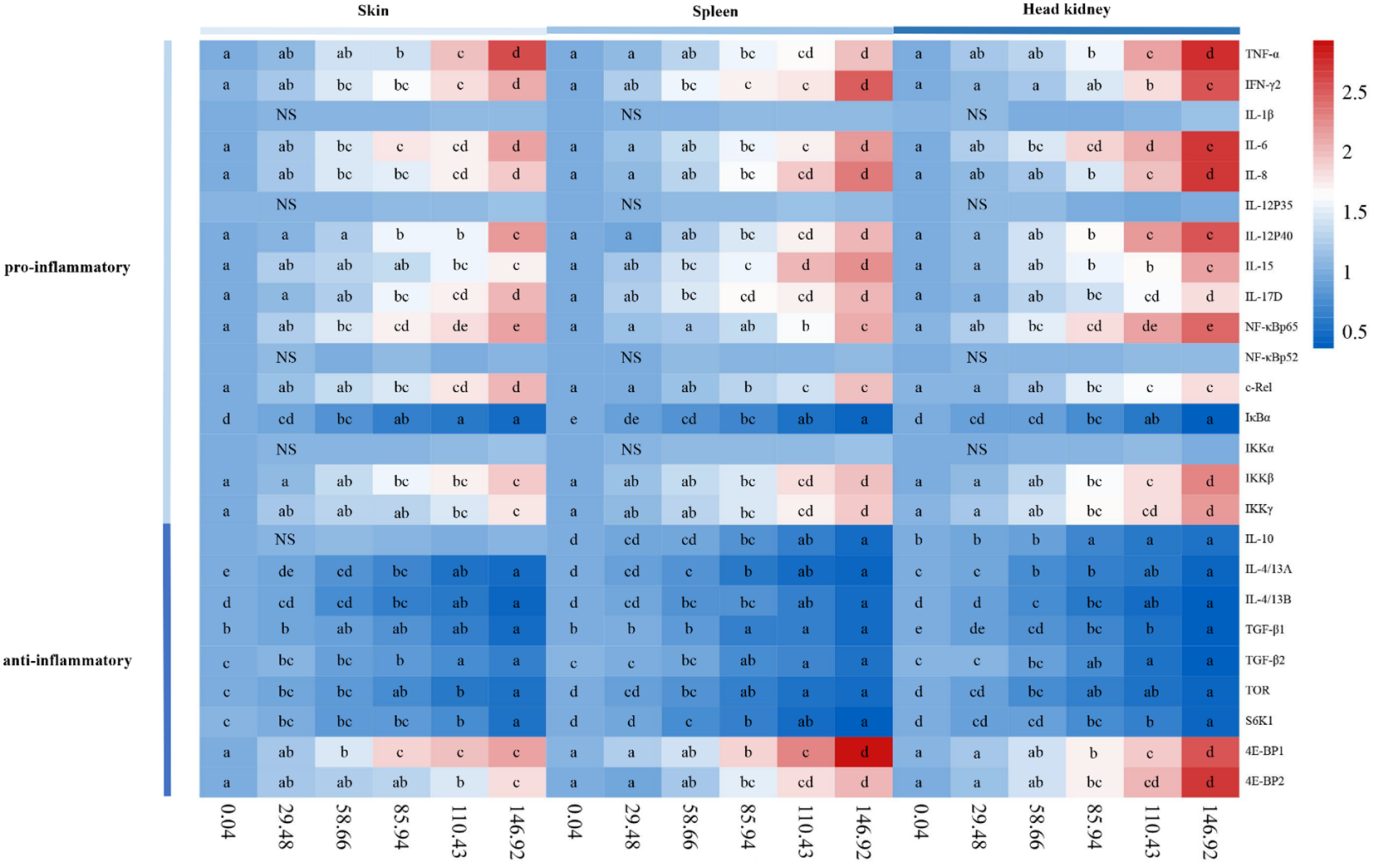
Heat-map of AFB1-changed mRNA levels of pro-inflammatory cytokines, anti-inflammatory cytokines, and inflammatory response related signal molecules in skin, spleen and head kidney of grass carp after *A. hydrophila* infection. n=6 (six fish in each group). Different letters indicate significant differences (P < 0.05).

The pro-inflammatory cytokines were shown in **Figure 4**. In the skin, the gene expression of *IL-8*, *IL-15*, *IL-6*, *IL-17D*, tumor necrosis factor α (*TNF-α*), interferon γ2 (*IFN-γ2*), and *IL-12p40* were significantly upregulated with the AFB1 levels up to 58.66, 110.43, 58.66, 85.94, 85.94, 58.66, and 85.94 μg/kg diet (*P* < 0.05), respectively. In the spleen, the gene expression of the above pro-inflammatory cytokines was significantly upregulated with the AFB1 levels up to 85.94, 58.66, 85.94, 58.66, 85.94, 58.66, and 85.94 μg/kg diet (*P* < 0.05), respectively. In the head kidney, the gene expression of the above pro-inflammatory cytokines was significantly upregulated with the AFB1 levels up to 85.94, 85.94, 58.66, 85.94, 85.94, 110.43, and 85.94 μg/kg diet (*P* < 0.05), respectively. Interestingly, in the skin, spleen, and head kidney, *IL-1β* and *IL-12p35* mRNA expression were insignificantly (*P*>0.05) at any levels of AFB1.

The anti-inflammatory cytokines were shown in **Figure 4**. In the skin, *TGF-β1*, *IL-4/13A*, *TGF-β2*, and *IL-4/13B* gene expression were significantly downregulated with the AFB1 levels up to 146.92, 58.66, 85.94, and 85.94 μg/kg diet (*P* < 0.05), respectively. In the spleen, the above anti-inflammatory cytokines gene expression was significantly downregulated with the AFB1 levels up to 85.94, 58.66, 58.66, 85.94, and 85.94 μg/kg diet (*P* < 0.05), respectively. In the head kidney, the transcription levels of *IL-10*, *IL-4/13A*, *IL-4/13B*, *TGF-β1*, and *TGF-β2* were significantly downregulated (*P* < 0.05), when the AFB1 dose reached 85.94, 58.66, 58.66, 58.66, and 85.94 μg/kg diet, respectively. Interestingly, the transcription level of *IL-10* has not been changed at any levels of AFB1 in the skin, spleen, and head kidney of grass carp.

The inflammatory signaling molecules were shown in **Figure 4**. In the skin, *c-Rel*, *NF-κBp65*, *IKK-β*, *IKK-γ*, *4E-BP1*, and *4E-BP2* genes expression were significantly upregulated when the AFB1 dose reached 85.94, 58.66, 85.94, 110.43, 58.66, and 110.43 μg/kg diet (*P* < 0.05), respectively. *IκBα*, *TOR*, and *S6K1* gene expression were significantly downregulated when the AFB1 levels were up to 58.66, 85.94, and 110.43 μg/kg diet (*P* < 0.05), respectively. In the spleen, *NF-κBp65*, *c-Rel*, *IKK-β*, *IKK-γ*, *4E-BP1*, and *4E-BP2* genes expression were upregulated when the AFB1 dose reached 110.43, 85.94, 85.94, 85.94, 85.94, and 85.94 μg/kg diet (*P* < 0.05), respectively. But *IκBα*, *TOR*, and *S6K1* gene expression were all significantly downregulated when the AFB1 dose reached 58.66 μg/kg diet (*P* < 0.05). In the head kidney, *NF-κBp65*, *c-Rel*, *IKK-β*, *IKK-γ*, *eIF4E-binding protein 1* (*4E-BP1*), and *4E-BP2* genes expression were significantly upregulated when the AFB1 dose reached 58.66, 85.94, 85.94, 85.94, 85.94, and 85.94 μg/kg diet (*P* < 0.05), respectively. While *IκBα*, *TOR*, and *S6K1* transcription levels were significantly reduced, when the AFB1 levels were up to 85.94, 58.66, and 85.94 μg/kg diet (*P* < 0.05), respectively. Interestingly, *NF-κBp52* and *IKKα* gene expression had no significant effect on the immune organs at any levels of AFB1.

### Protein expression levels of nuclear factor-κB p65 (NF-κB p65) and phosphorylation of TOR in skin, spleen, and head kidney of grass carp

**Figure 5:**
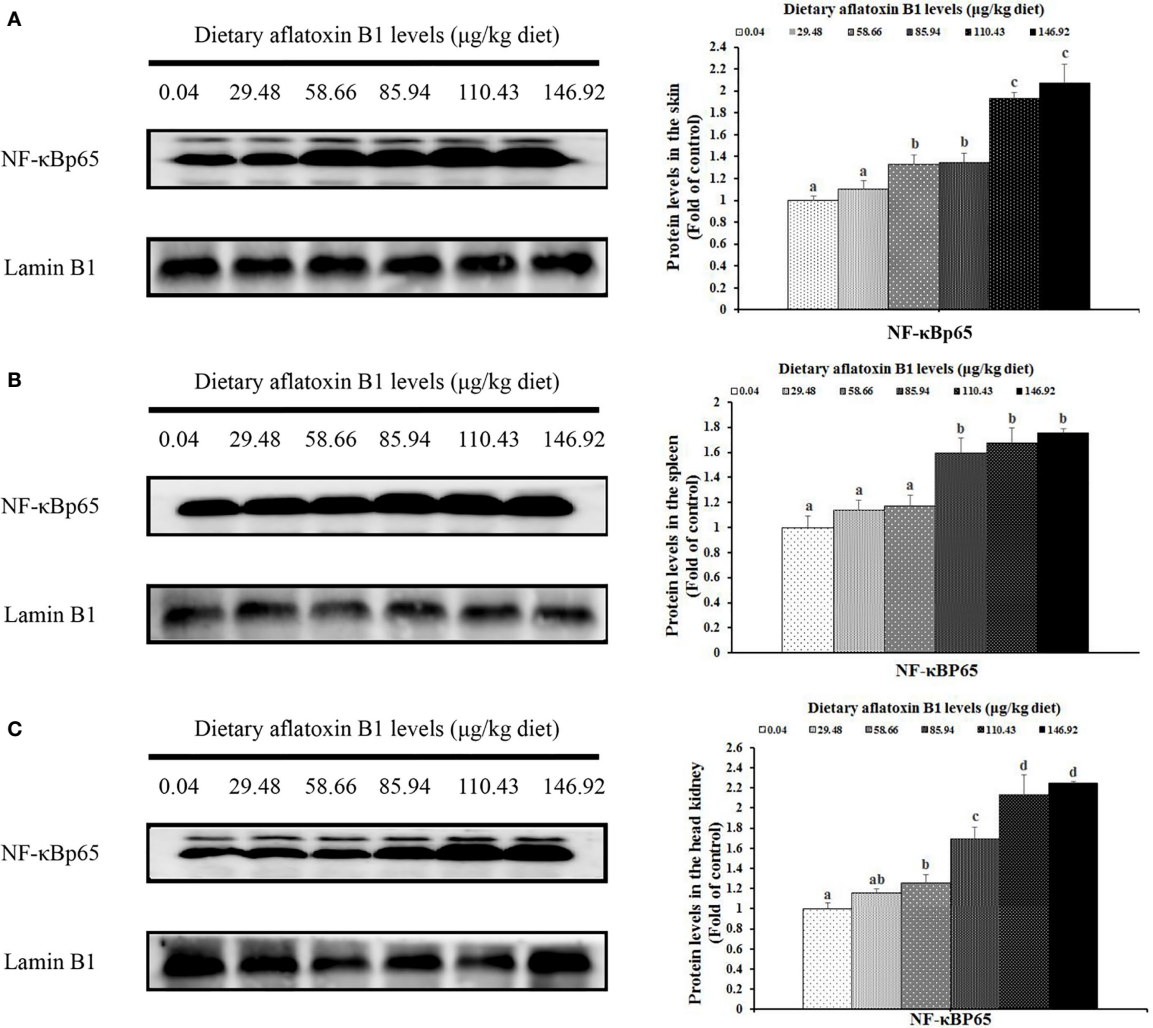
The protein levels NF-kB p65 in the skin **(A)**, spleen **(B)** and head kidney **(C)** of grass carp after infection of *A hydrophila*. n=6 (six fish in each group). Different letters indicate significant differences (P < 0.05).

**Figure 6:**
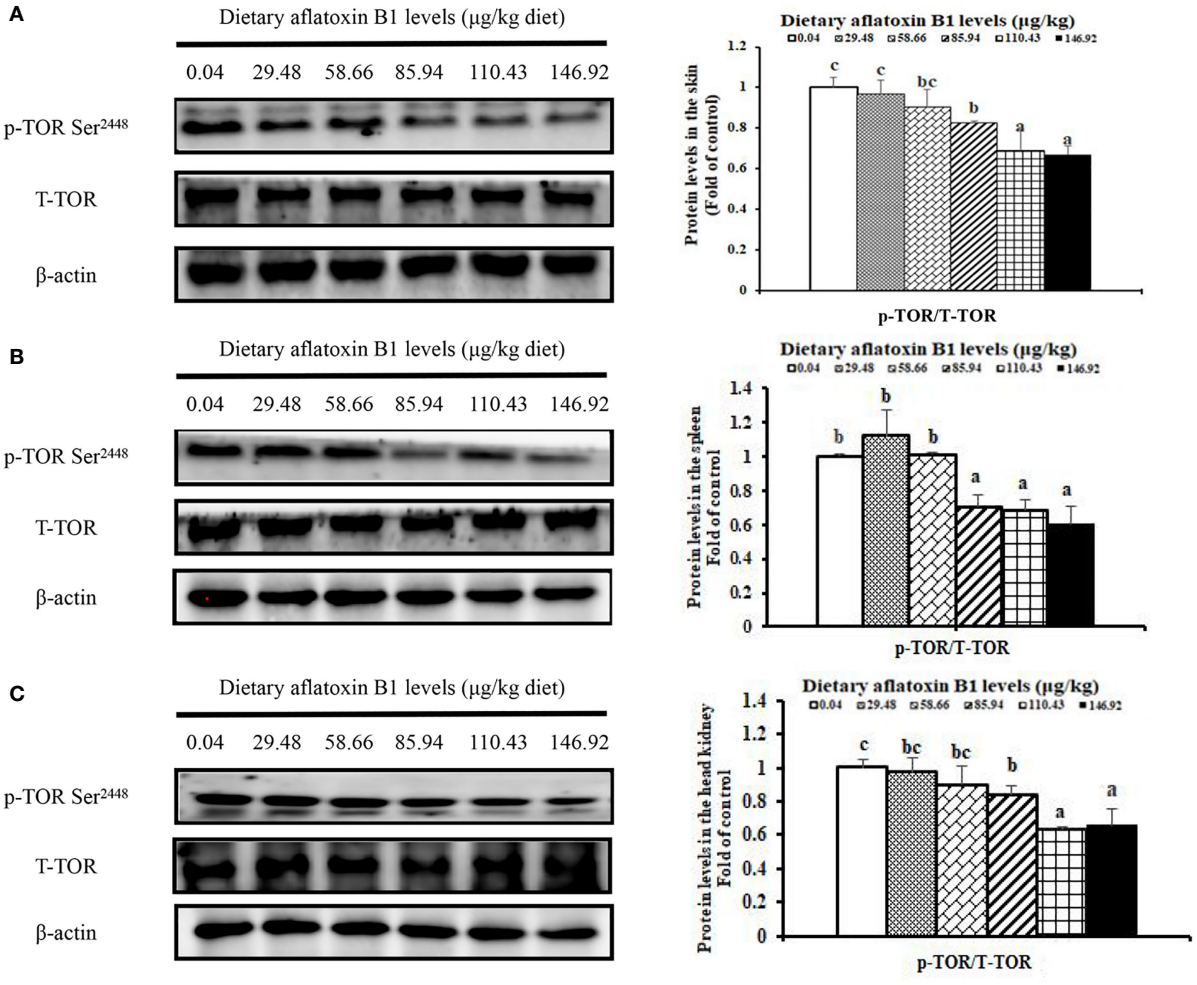
The protein levels phosphorylation of TOR at Ser2448 in the skin **(A)**, spleen **(B)** and head kidney **(C)** of grass carp after infection of *A hydrophila*. n=6 (six fish in each group). Different letters indicate significant differences (P < 0.05).

In **Figure 5**, dietary AFB1 led to the decrease of nuclear NF-κB p65 in a dose-dependent manner in the immune organs of grass carp. In the skin, spleen, and head kidney, the levels of NF-κB p65 protein were significantly upregulated when the AFB1 levels reached 58.66, 85.94, and 58.66 μg/kg diet (*P* < 0.05), respectively. In **Figure 6**, dietary AFB1 led to the decrease of the protein in a dose-dependent manner in the grass carp skin, spleen, and head kidney. Western blot analysis indicated that grass carp had significantly decreased both the levels of p-TOR Ser^2448^ and T-TOR protein when the dietary AFB1 levels reached 85.94 μg/kg diet (*P* < 0.05) in the skin, spleen, and head kidney, respectively. Both the levels of p-TOR Ser^2448^ and T-TOR protein reached the lowest levels in the 146.92 μg/kg diet groups in the skin, spleen, and head kidney.

## Discussion

Using the same growth test as our previous study, we conducted research as part of a larger project to evaluate the immune function of AFB1 on the immune organs (skin, spleen, and head kidney) of grass carp (6). It has been confirmed that dietary AFB1 inhibited fish growth and disrupted the physical barrier of grass carp spleen and head kidney in our previous study (6). However, in addition to physical barriers, fish immune organ health also depends on immune barriers (37). It was the primary aim of this study to determine whether AFB1 affected inflammation in fish immune organs (head kidney, spleen and skin) after infection with *A. hydrophila*.

### AFB1 attenuated immunological parameters of immune organs

As is well known that the function of the immune barrier of fish is mediated by immune substances, which include ACP and LZ activities, C3, C4, and immunoglobulins contents, and the antibacterial peptides gene expression (38). As an important component of innate immune response, ACP and LZ have excellent bactericidal effects, whereas complement system is involved in the recognition and elimination of pathogens in fish (39, 40). In addition, antimicrobial peptides are also important components of fish innate immunity, mainly including β-defensin1, LEAP-2A, LEAP-2B, etc. (14). Our results found that AFB1 decreased the activities of LZ, ACP, the contents of C3, C4, and IgM, and downregulated *β-defensin1*, *LEAP-2A*, *LEAP-2B*, and *Mucin2* gene expression in skin, spleen, and head kidney of grass carp. Similarly, Sahoo et al. (35) found that AFB1 decreased LZ activity in rohu serum, and another research also confirmed that AFB1 significantly decreased the contents of immune components (e.g. C3, C4) in juvenile turbot serum (21), which were consistent with our results, suggesting that AFB1 could suppress the immune function of the skin, spleen, and head kidney partly referring to decrease immune components in fish. The reason for the inhibition of AFB1 on immunological parameters may be attributed to the reduction of the number of immune cells. It is well known that lymphocytes could produce immune components (40), such as LZ, IL-4, and IL-8, etc. A study observed that AFB1 reduced lymphocyte proliferation in rainbow trout (41). The present study showed that AFB1 could decrease the content of LZ. As far as we know, lysozyme is derived from monocytes and neutrophils, which were a large number of distributions in the spleen and head kidney. Although the above evidence has been presented, a large number of experimental data are still needed to further confirm. Furthermore, it should be noted that, the spleen and head kidney of grass carp did not exhibit any changes in *hepcidin* transcription levels due to AFB1. The reason may be partially related to the *IL-1β* mRNA levels. Research on murine exhibited that *IL-1β* participates in the regulation of *hepcidin* in primary hepatocytes (42). Coincidentally, our results found that AFB1 had no impact on *IL-1β* mRNA expression. Nevertheless, the mechanisms involved in this process need to be studied in greater detail.

### AFB1 attenuated inflammatory response of immune organs

Cytokines-mediated inflammation response is a hallmark of immune system up-regulation (43, 44). It is largely acknowledged that the inflammation could be aggravated by increasing the transcription levels of pro-inflammatory cytokines and decreasing the transcription levels of anti-inflammatory cytokines in fish, which were regulated by NF-κB and TOR (45, 46). Generally, adverse external stress can usually lead to drastic changes in immune homeostasis, mainly manifested as dynamic changes in inflammatory cytokines. The current research demonstrated that dietary AFB1 increased the pro-inflammatory cytokines transcription levels and decreased the anti-inflammatory cytokines transcription levels in the skin, spleen, and head kidney of grass carp. Similarly, one research demonstrated that dietary AFB1 increased the transcription levels of TNF-α and IL-1β in rainbow trout intestinal (5), another study found that dietary AFB1 significantly increased IFN-γ content in the broiler jejunum mucosa (23); in addition, dietary AFB1 downregulated *IL-10* mRNA expression in chicken livers (47), which were consistent with the above results. Combining the above mentioned evidences, AFB1 induced inflammatory responses in fish immune organs has been observed. Based on our assumptions, there might be a connection between AFB1 and immune cells. A variety of inflammatory cytokines are released by macrophages and immune cells during the inflammatory process (48). The *in vitro* study revealed that AFB1 inhibits the growth of murine macrophages and decreases various secretory cytokines found in these cells (49). Furthermore, we observed two interesting findings. Firstly, AFB1 had not changed the transcription levels of *IL-1β* in the skin, spleen, and head kidney of grass carp. The possible reason may be that the contact time and dose of AFB1 to grass carp were insufficient. Similarly, Dugyala and Sharma et al. (50) found that 0.145 mg AFB1/kg body weight induced *IL-6* and *TNF-*α higher expression in rat peritoneal macrophages at 2 weeks, nevertheless Rossano et al. (51) found that 1.0 pg/ml AFB1 downregulated *IL-6* and *TNF-*α mRNA expression in and human monocytes at 2h and 12h. The possible reason accounting for the different results is that the time and dose of AFB1 exposed to macrophages were different. This might support our hypothesis, but the clear mechanisms need further investigation. Secondly, in the present experiment, AFB1 only upregulated *IL-12p40* rather than *IL-12p35* in the skin, spleen, and head kidney of grass carp. It was reported that the stable expression of different subtypes of mRNA may be different in different tissues (52). Pandit et al. (53) found that the mRNA of *IL-12p40* is high expression in immune tissues (such as the spleen and head kidney) of grass carp during *A. hydrophila* infection, while the mRNA of *IL-12p35* is mostly expressed in the central nervous system. This might support our hypothesis. Its exact mechanism of action needs to be determined through further research.

### AFB1 modulates immune response related signaling pathways

As we have known, the activation of the IKK complex promotes the degradation of IκBα and then activates NF-κB, whose activation could increase the pro-inflammatory cytokines transcription levels in humans (54). In humans, the level of nuclear NF-κB protein could be a symbol of activating NF-κB signaling (55). In the current work, we observed that AFB1 upregulated the mRNA levels of nuclear NF-κB protein in grass carp skin, spleen, and head kidney. Correlation analyses displayed that *IFN-γ2*, *TNF-α*, *IL-6*, *IL-8*, *IL-12p40*, *IL-15*, and *IL-17D* gene expression were positively correlated with the NF-κBp65 protein levels (**Table 2**), suggesting that AFB1 aggravated spleen and head kidney inflammation partially related to activation of NF-κBp65 signaling in grass carp. The reason for the activation of NF-κB signaling by AFB1 was still not clarified in the immune organs of fish. Probably two reasons contributed to this. Firstly, this is partially related to IkBα, IKKβ, and IKKγ. Studies suggested that IKKβ, γ could activate NF-κB nuclear translocation, and IκBα could inhibit NF-κB nuclear translocation (55). Our results found that AFB1 upregulated *IKKβ*, and *IKKγ* genes expression and downregulated *IκB*α gene expression in the skin, spleen, and head kidney of grass carp, which supports our hypothesis. Secondly, it is might associated with the mRNA levels of the *NF-κB*. Takahashi et al. (56) found that upregulating the *NF-κB* mRNA levels could increase the levels of the NF-kBp65 protein level. Our results indicated that AFB1 upregulated the *NF-κB p65* gene expression in grass carp skin, spleen, and head kidney, which is consistent with the results of the protein expression from the current study. Interestingly, the results from our study found that AFB1 had not changed *NF-*κ*Bp52* transcription level in grass carp skin, spleen, and head kidney. The possible reason is linked to the stably *IKKα* gene expression. It was shown that *NF-κBp52* could be specifically activated by *IKKα* in mammalian cells (57). The current results showed that AFB1 had not changed the *IKKα* transcription level in the skin, spleen, and head kidney of grass carp, which supported our assumption.

**Table 2:** Correlation coefficient of parameters in the immune organs of grass carp^1^.

Independent parameters	Dependent parameters	Spleen		Head kidney	
		Correlation coefficients	P	Correlation coefficients	P
NF-κB p65 protein level	TNF-α	+0.970	<0.01	+0.957	<0.01
	IFN-γ2	+0.885	<0.01	+0.920	<0.05
	IL-6	+0.935	<0.01	+0.969	<0.01
	IL-8	+0.951	<0.01	+0.936	<0.01
	IL-12P40	+0.936	<0.01	+0.983	<0.01
	IL-15	+0.941	<0.01	+0.943	<0.01
	IL-17D	+0.947	<0.01	+0.971	<0.01
c-Rel mRNA level	TNF-α	+0.937	<0.01	+0.955	<0.01
	IFN-γ2	+0.968	<0.01	+0.933	<0.01
	IL-6	+0.972	<0.01	+0.986	<0.01
	IL-8	+0.961	<0.01	+0.939	<0.01
	IL-12P40	+0.960	<0.01	+0.983	<0.01
	IL-15	+0.928	<0.01	+0.978	<0.01
	IL-17D	+0.914	<0.01	+0.988	<0.01
IκBα mRNA level	NF-κB p65 protein level	-0.965	<0.01	-0.978	<0.01
	c-Rel	-0.926	<0.01	-0.982	<0.01
IKKβ mRNA level	NF-κB p65 protein level	+0.940	<0.01	+0.970	<0.01
	IκBα	-0.992	<0.01	-0.996	<0.01
IKKγ mRNA level	NF-κB p65 protein level	+0.944	<0.01	+0.972	<0.01
	IκBα	-0.989	<0.01	-0.995	<0.01
p-TOR protein level	IL-4/13A	+0.964	<0.01	+0.932	<0.01
	IL-4/13B	+0.870	<0.01	+0.963	<0.05
	IL-10	+0.941	<0.01	+0.959	<0.01
	TGF-β1	+0.980	<0.01	+0.957	<0.01
	TGF-β2	+0.973	<0.01	+0.954	<0.01

^1^“+” means positive correlation; “-” means negative correlation.

Studies in mammalian have found that activation of mTOR signaling promotes the production of anti-inflammatory cytokines (e.g., IL-10, TGF-β1). According to previous study, S6K1 and 4E-BP are key signaling molecules downstream of mTOR protein (46). A study has revealed that phosphorylation of TOR Ser2448 can reflect TOR signaling activation in Jian carp (*Cyprinus carpio* var. *Jian*) (58). Our results demonstrated that AFB1 reduced p-TOR Ser2448 transcription level in the skin, spleen, and head kidney of grass carp, which demonstrated that AFB1 inhibited the activation of TOR signaling. Correlation analyses displayed that *TGF-β1*, *TGF-β2*, *IL-10*, *IL-4/13A*, and *IL-4/13B* gene expression were positively correlated with the p-TOR Ser2448 (**Table 2**), suggesting that AFB1 aggravated spleen and head kidney inflammation partly be associated with the inhibition of TOR signaling in fish. The reason for the inhibition signaling of TOR by AFB1 may be related, in part to TOR transcription and translation. Kew et al. (57) indicated that the level of p-TOR Ser2448 protein was in contact with its transcription and translation. Our present result showed that AFB1 could decrease *TOR* transcription level in the grass carp skin, spleen, and head kidney, which support our hypothesis. Our results indicated that AFB1 aggravated inflammation might related to the activation of NF-κBp65 signaling and inhibition of TOR signaling.

### AFB1 safe upper dose for grass carp

Intensive farming poses health-threatening to fish. However, the health of fish is associated with its immunity. The increase in morbidity is used to assess the adverse effects of harmful substances on the body. ACP and LZ activities, as important immune indicators, often used to evaluate the health of fish immune organs (59). Therefore, in order to maintain the skin, spleen, and head kidney health of grass carp, we selected the dose with no significant effect of AFB1 on ACP and LZ as the safe upper dose. Finally, we determined that AFB1 not exceeding 29.48 μg/kg diet could maintain the health of immune organs.

## Conclusion

**Figure 7:**
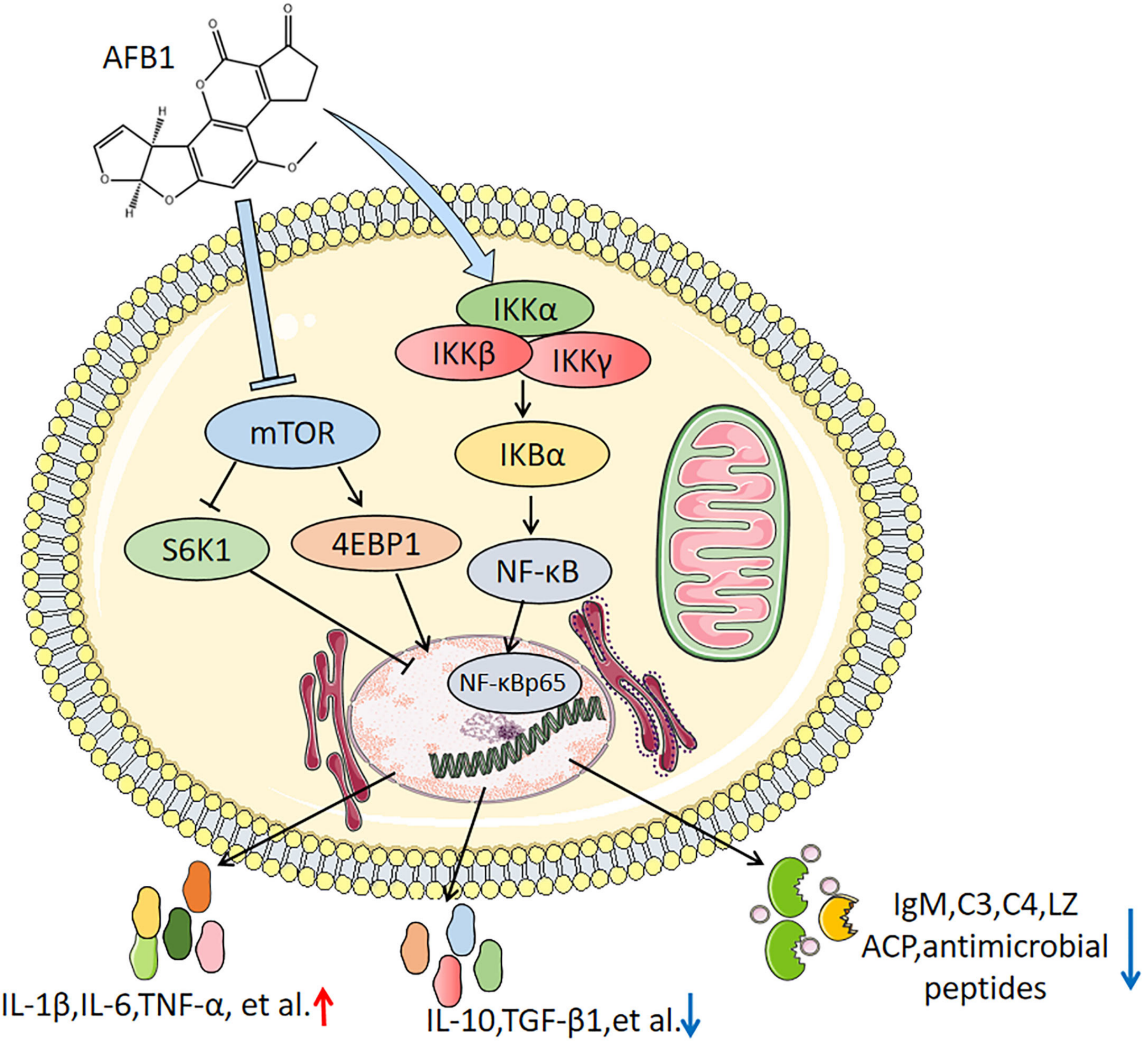
The mechanism of AFB1 on immune function of immune cells in the skin, spleen and head kidney of grass carp.

In conclusion, as shown in **Figure 7**, the results of our study demonstrate that AFB1 destructed immune competent of grass carp skin, spleen, and head kidney (1). Dietary AFB1 decreased C3, C4, and IgM contents, LZ and ACP activities, and downregulated *β-defensin1*, *Mucin2*, *LEAP-2A*, and *LEAP-2B* gene expression in skin, spleen, and head kidney of grass carp (2). Dietary AFB1 promoted inflammatory responses related to the activation of [(*IKKβ* and *IKKγ* rather than *IKKα*)/*IκBα*/*NF-κB* (*p65* and *c-Rel* rather than *p52*) to upregulate the pro-inflammatory cytokines genes expression (except *IL-1β* and *IL-12p35*), and inhibition of [*TOR*/(*S6K1*, *4E-BP1*)] signaling to downregulate the anti-inflammatory cytokines genes expression in grass carp skin, spleen, and head kidney. Moreover, the content of AFB1 in the grass carp diet should not exceed 29.48 μg/kg diet according to the activities of LZ and ACP.

## Data availability statement

The original contributions presented in the study are included in the article/**Supplementary Material**. Further inquiries can be directed to the corresponding authors.

## Ethics statement

The animal study was reviewed and approved by Sichuan Agricultural University Animal Care Advisory Committee.

## Author contributions

X-NH: Writing-Original draft preparation, Formal analysis, Investigation. Z-ZZ: Formal analysis, Investigation. LF: Data Curation. W-DJ, PW: Methodology. YL, JJ: Validation. S-YK, LT: Resources. X-QZ: Conceptualization, Writing-Reviewing and Editing, Project administration. All authors contributed to the article and approved the submitted version.

## Funding

This research was financially supported by National Key Research and Development Program of China (2019YFD0900200, 2018YFD0900400), National Natural Science Foundation of China for Outstanding Youth Science Foundation (31922086), Supported by Earmarked found for China Agriculture Research System (CARS-45), and supported by Sichuan Science and Technology Program (2019YFN0036).

## Acknowledgments

The authors would like to express theirsincere thanks to the personnel of these teams for theirkind assistance.

## Conflict of interest

The authors declare that the research was conducted in the absence of any commercial or financial relationships that could be construed as a potential conflict of interest.

## Publisher’s note

All claims expressed in this article are solely those of the authors and do not necessarily represent those of their affiliated organizations, or those of the publisher, the editors and the reviewers. Any product that may be evaluated in this article, or claim that may be made by its manufacturer, is not guaranteed or endorsed by the publisher.
