# Supplementary material for: Dietary Aflatoxin B1 attenuates immune function of immune organs in grass carp (Ctenopharyngodon idella) by modulating NF-κB and the TOR signaling pathway
Source: Front Immunol. 2022 Oct 18;13:1027064. doi: 10.3389/fimmu.2022.1027064 (PMC9623247; doi:10.3389/fimmu.2022.1027064)
Supplement: Supplementary file 1 [file Table_1.docx]

**Supplementary materials**

**Supplementary Table 1.** The information of antibodies (Western blot)

| Indices | Host | Source | Catalog No. | Dilution for WB |
| --- | --- | --- | --- | --- |
| NF-κB p65 | Rabbit | Affinity (Golden, Colorado, USA) | AF5006 | 1:750 |
| LaminB1 | Rabbit | Affinity (Golden, Colorado, USA) | AF5161 | 1:1000 |
| p-TOR Ser 2448 | Rabbit | Affinity (Golden, Colorado, USA) | AF3308 | 1:1000 |
| Total TOR | Rabbit | Affinity (Golden, Colorado, USA) | AF6308 | 1:1000 |
| β-actin | Rabbit | Affinity (Golden, Colorado, USA) | AF7018 | 1:3000 |
